# Biomaterial-Assisted Regenerative Medicine

**DOI:** 10.3390/ijms22168657

**Published:** 2021-08-12

**Authors:** Teruki Nii, Yoshiki Katayama

**Affiliations:** 1 Department of Applied Chemistry, Faculty of Engineering, Kyushu University, 744 Motooka, Nishi-ku, Fukuoka 819-0395, Japan; 2 Graduate School of Systems Life Sciences, Kyushu University, 744 Motooka, Nishi-ku, Fukuoka 819-0395, Japan; 3 Center for Future Chemistry, Kyushu University, 744 Motooka, Nishi-ku, Fukuoka 819-0395, Japan; 4 International Research Center for Molecular Systems, Kyushu University, 744 Motooka, Nishi-ku, Fukuoka 819-0395, Japan; 5 Department of Biomedical Engineering, Chung Yuan Christian University, 200 Chung Pei Rd., Chung Li 32023, Taiwan

**Keywords:** regenerative medicine, biomaterials, cell transplantation, tissue engineering, drug research

## Abstract

This review aims to show case recent regenerative medicine based on biomaterial technologies. Regenerative medicine has arousing substantial interest throughout the world, with “The enhancement of cell activity” one of the essential concepts for the development of regenerative medicine. For example, drug research on drug screening is an important field of regenerative medicine, with the purpose of efficient evaluation of drug effects. It is crucial to enhance cell activity in the body for drug research because the difference in cell condition between in vitro and in vivo leads to a gap in drug evaluation. Biomaterial technology is essential for the further development of regenerative medicine because biomaterials effectively support cell culture or cell transplantation with high cell viability or activity. For example, biomaterial-based cell culture and drug screening could obtain information similar to preclinical or clinical studies. In the case of in vivo studies, biomaterials can assist cell activity, such as natural healing potential, leading to efficient tissue repair of damaged tissue. Therefore, regenerative medicine combined with biomaterials has been noted. For the research of biomaterial-based regenerative medicine, the research objective of regenerative medicine should link to the properties of the biomaterial used in the study. This review introduces regenerative medicine with biomaterial.

## 1. Introduction

**Figure 1:**
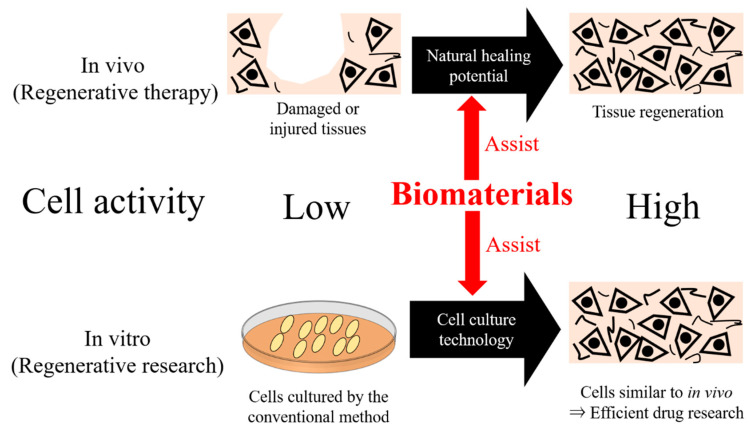
Biomaterials are promising methods to enhance the biological function of cells in vivo and in vitro, leading to the realization of regenerative medicine. In vivo, tissue regeneration can be achieved when the activity of cells in the damaged tissues enhances. Furthermore, if the cell activity is high enough in cell culture, similar to in vivo, it is possible to effectively predict the drug effect in a preclinical or clinical study. Thus, biomaterial-assisted regenerative medicine has been recently identified as a promising approach.

Regenerative medicine is one of the most attractive fields in recent biomedical engineering. For the development of regenerative medicine, it is essential to enhance cell activity. For example, in the damaged or injured tissues, the natural healing potential is too low for cells to migrate, proliferate, and differentiate. If damaged cells’ natural healing potential can be enhanced by using scientific technology, “patient-friendly” tissue regeneration could be achieved. For in vitro research, living cells should be used with satisfying functions and viability [1]. Cells are usually cultured in a dish that is mainly composed of polystyrene—this culture condition is artificial, and the environmental situation is quite different from the original tissues. The difference in the cell condition leads to low cell activity compared to in vivo, as cells in the body interact well with other cells or extracellular matrix (ECM), resulting in enhanced cell activity in their differentiation [2], proliferation [3], metabolism [4], or cytokine secretion [5,6]. The drug effect found in vitro drug screening conditions is not always the same as in a preclinical or clinical study because of the difference in the cell condition or activity [7,8,9]. If the cells with high activity are used in drug screening, the efficient evaluation of drug effects could be achieved. Thus, for the advancement of regenerative medicine, it is essential to enhance the function or activity of cells both in vivo and in vitro (Figure 1).

The use of biomaterials is one of the most effective methods to enhance cell activity (Figure 1). The American National Institute for Health (NIH) standard definition of biomaterials is “any substance or combination of substances, other than drugs, synthetic or natural in origin, which can be used for any period of time, which augments or replaces partially or totally any tissue, organ of function of the body, in order to maintain or improve the quality of life of the individual” [10]. In particular, biomaterials composed of ECM components can be useful to enhance cell activity [11,12], because it is well known that ECM enables cells to enhance viability or function [13,14,15].

Polymeric biomaterials, one of the essential biomaterials, can be classified into natural biomaterials and synthetic biomaterials. Natural biomaterials are composed of polysaccharides (chitosan, alginate, or hyaluronic acid) or peptide (collagen or gelatin), while polyethylene glycol, poly(lactic acid), or poly(lactic-*co*-glycolic acid) are well known synthetic polymers. The advantage of natural biomaterials is their high biocompatibility, as the endogenous enzymes can degrade the biomaterials. On the other hand, synthetic polymers have flexibility in their structural design to modify cell functions easily [12]. Therefore, it is essential to understand the properties of each biomaterial and select the appropriate biomaterials considering the purpose of the studies.

The objective of this review is to show recent regenerative medicine approaches based on biomaterial technologies, because it is essential to understand the properties of each biomaterial and select the appropriate biomaterial for each potential regenerative medicine. This review introduces recent representative studies of regenerative medicine, such as tissue engineering or drug research, using several biomaterials.

## 2. Regenerative Medicine Combined with Biomaterials

The basic information of several biomaterials and biomaterials-assisted regenerative medicine are introduced in Table 1, which summarizes recent regenerative medicine combined with biomaterials. We collected the studies including three keywords: research using representative biomaterials, research to show the result of enhancing biological function, and research reported in the last five years. As representative natural biomaterials, collagen, gelatin, alginate, chitosan, silk fibroin, agarose, and Matrigel were selected. In contrast, poly(lactic acid) and poly(lactic-*co*-glycolic acid) for synthetic biomaterials were introduced in this review. Although there are other synthetic polymers, the two polymers were selected because of the medical application, availability, and ease to handle.

### 2.1. Collagen

Collagen is the most abundant protein in the body and supports mechanical and structural conditions [80]. Collagen is mainly composed of glycine, proline, or hydroxyproline. A hydrogen bond forms the collagen triple helix. The main types of collagen are type I (skin, tendon, or bone), II (cartilage), III (skin vessel), and IV (basement membrane) [81]. Due to the abundant existing ratio, collagen is an essential protein for cells to enhance cell function [82]. For example, collagen crosslinking and stiffening promotes the aggregates of breast cancer [83], therefore, collagen is widely used as a material for the tissue engineering of skin [84], bone [16,17,85], cartilage [18,86], blood vessels [87], muscle [19], or cancer [20,21,22]. For example, when mesenchymal stem cells (MSC) are cultured on Type Ⅰ collagen gels, the osteoblast marker, such as alkaline phosphatase activity, collagen synthesis, or osteocalcin gene, is enhanced [85]. 

The composite of collagen and biphasic calcium phosphate nanoparticles with a controlled release of dexamethasone has also been prepared. The material enables efficient bone tissue regeneration from MSC in vitro. High bone regeneration is observed when the materials are injected into the dorsal of athymic nude mice [16]. Heo et al. prepared collagen hydrogel encapsulating multicellular spheroids of MSC and human umbilical vein endothelial cells. The spheroid showed cell spreading, proliferation, osteogenic differentiation, and pre-vascular network in the hydrogel because collagen gel provides cells a suitable environment [17]. Collagen material is specially selected in the three-dimensional culture of cancer cells to evaluate migration, invasion, or metastasis because the cancer cells prefer to migrate into type Ⅰ collagen in vivo [88]. There is a report that the degree of collagen fiber alignment or the fibril bending stiffness of the collagen matrix affects the behavior of breast cancer cells [23]. Moreover, when lung or pancreatic cells were co-cultured with fibroblasts into collagen gels, cancer cells migrated efficiently [22]. Recently, to investigate the reaction of T cells under the tumor microenvironment, T cells are cultured with collagen gels of different densities [89]. Indeed, collagen is the most representative biomaterial. Researchers will continue studying the effect of collagen material on biological function in vitro and in vivo.

### 2.2. Gelatin

Collagen material is effective because collagen is a prominent ECM component. However, there is a limitation of collagen as a biomaterial due to its low solubility in water and biological activities. Gelatin, a denatured form of collagen, as water-soluble material, is often used in biomedical approaches [90]. Gelatin hydrogels can permeate the oxygen or nutrient because of the high water content [91,92]—this permeability is effective in regenerative medicine. For example, cells present in the center of spheroids or the center layer of the multilayer cell sheet are dead with hypoxia [93]. Tabata et al. have incorporated the gelatin hydrogels into the spheroids or between each cell sheet to tackle this problem. This method enables the culture of the spheroids or cell sheets for an extended period [24,26,27,28]. The gelatin hydrogels not only permeate oxygen but also contain growth factors [25,29,30]. As mentioned above, growth factors are essential to enhance cell activity. When the gelatin hydrogels containing basic fibroblasts growth factors (bFGF) were injected into damaged tissues, effective vascularization was observed, resulting in tissue regeneration [94,95]. There are two advantages of the gelatin hydrogel microspheres; one is the drug release mechanism. The growth factors are released from the materials not by diffusion but by the degradation of materials with degradation enzymes, which means the drug is released sustainably when injecting the gelatin materials into damaged tissues. The other is the eventual disappearance of the gelatin hydrogel microspheres. To repair the damaged or injured tissues, cells near the damaged tissues should migrate, proliferate, and differentiate. The material-remaining leads to the physical impairment of tissue regeneration [1]. Therefore, the materials injected must disappear during tissue regeneration. The gelatin hydrogel microspheres disappear eventually, and the degradation speed can be changed and modified by the chemical crosslinking condition, responding to the damage level [95].

The gelatin hydrogel microspheres are also effective in drug discovery. Cancer invasion is one of the issues to be solved [96]. The cancer invasion model would be effective in anti-cancer drug screening. It is well known that the interaction between cancer cells and stromal cells, especially cancer-associated fibroblasts (CAF), promotes cancer invasion [97]. 3D CAF aggregates incorporating gelatin hydrogel microspheres capable of drug release are prepared to mimic the cancer invasion. The CAF aggregates increase the invasion rate of cancer cells [6,31,32], herefore, the characteristics of gelatin hydrogel microspheres, such as oxygen permeability, drug release mechanisms, or eventual disappearance, are desirable for building a cancer tissue model for the screening of anti-cancer drugs.

In addition to hydrogels, gelatin hydrogel nonwoven fabrics have also been recently reported. The mechanical properties of the gelatin hydrogel nonwoven fabrics are strong enough to be handled in swollen conditions [98,99,100]. When multilayered cell sheets are cultured with the gelatin hydrogel nonwoven fabric, the transfer time of the cell sheets is improved. In addition, glucose consumption or adenosine triphosphate (ATP) production of multilayered cell sheets enhances by formulating with the gelatin hydrogel nonwoven fabrics between each cell sheet [99].

Moreover, cationized gelatin nanospheres incorporating imaging probes to detect mRNA have been recently prepared [101]. For cell transplantation, the non-invasive technology to detect the cellular localization and distribution or biological function after transplantation is needed. The nanospheres aim to visualize cellular function, such as apoptosis [102], macrophage phenotypes [103], or cell proliferation ability [104].

### 2.3. Alginate

Alginate, a copolymer of α-l-guluronic acid and β-d-mannuronic acid, is derived from seaweed [105]. Alginate is one of the attractive biomaterials in biomedical engineering because the molecular structure of alginate is similar to that of polysaccharides [106]. In addition, alginate gels are easily obtained by calcium or ferric ion at room temperature, and cell encapsulation into alginate gels has been extensively studied [107,108]. These gels, which incorporate cells, are effective for cell delivery to damaged tissues or in vitro cell research. In particular, stem cells [33,34,36,37,109,110,111], pancreatic-associated cells [38,39,112], or cancer cells [40,41] are often selected in the alginate encapsulation system. For example, An et al. encapsulated MSC into alginate gels. The systems showed efficient differentiation into osteoblast cells [34]. Mansouri et al. reported that alginate gels promote the differentiation into primordial germ cells of mouse embryonic stem cells [33]. Somo et al. prepared MIN6 of a pancreatic beta-cell line encapsulated into alginate gels to deliver to islets as a type 1 diabetes treatment [39], while Estrada et al. prepared alginate gels encapsulating breast cancer cells and fibroblasts cells to mimic the breast cancer microenvironment. As a result, the reduction in estrogen receptors, the loss of cell polarity, the increase of cancer cell migration, and enhanced angiogenesis potential were observed in this system [40]. Thus, a cell encapsulating system based on alginate gels is effective in tissue engineering or drug research.

Injectable gels for cell transplantation, taking advantage of cell encapsulating, have been reported. Injectable alginate and gelatin hydrogels containing cells are prepared by mixing alginate/gelatin solution and FeCl_3_ solution based on the physico-chemical interaction. They confirm the appropriate mixing ratio of alginate and gelatin for cell cytotoxicity, cell proliferation, and differentiation in vitro and in vivo. The strength of these injectable gels is to form gelation by not chemically or covalently crosslinking polymers but by physicochemical interaction. This crosslinking allows the gel to disintegrate quickly. It disappears a few days after the injection, while most injectable gels already reported take more than three weeks to disappear or even remain in the body due to the stable chemical crosslinking [35]. The disappearance characteristics of injectable alginate-based gels are suitable for tissue regeneration because the material that may remain for a long time sometimes causes the physical impairment of tissue regeneration. 

### 2.4. Chitosan

Chitosan, a copolymer of β-(1→4)-2-acetamido-D-glucose and β-(1→4)-2-amino-D-glucose units, is obtained by deacetylation of chitin [113]. The solubility of chitosan is much higher than that of chitin, which means that it is easy to handle. Chitosan is easily chemically modified because of the existence of β-(1,4) glycosidic bonds between d-glucosamine and N-acetyl-d-glucosamine [114]. Such modifications are used for imparting stiffness or low inflammatory induction property to chitosan [115]. Chitosan can also interact with negatively charged biomaterials [10,116]. Due to the low cost and versatility, chitosan is effective biomaterial as food packaging films [113,117], preservation of food [118] and drink [119], pharmaceutical science [120], cosmetics [121], or antibacterial agents [115]. In regenerative medicine, chitosan is often selected for blood vessels [42,43,44], cartilage [45,46,47,48,122,123,124], bone [49,50,125,126], the intervertebral disc [51,52,53,127,128], or skin [54,55,129] regeneration. For example, glycosaminoglycan (GAG) is essential to stimulate the formation of cartilage. The electronic interaction between the negatively charged GAG and chitosan is formed. GAG amount of cartilage cells with chitosan scaffold was higher 14 or 21 days after the transplantation [122]. In addition to the interaction with GAG, the structure of chitosan is close to that of GAG. Therefore, the chitosan scaffold can support cell culture because GAG is one of the most critical ECM components [11]. Chitosan scaffold enables human fibroblasts, endothelial cells, or keratinocytes to proliferate in vitro and in vivo [130]. As one trial for blood vessel regeneration, heparin and chitosan scaffold have been reported. Zhang et al. have prepared a multilayered vascular patch by alternately depositing the heparin-chitosan onto a polyurethane-coated decellularized platform via a layer-by-layer method. The vascular patch has a capacity for vascular tissue regeneration in vitro and in vivo [44]. Due to the biocompatibility and positively charged surface, chitosan is one of the most effective biomaterials for regenerative medicine.

### 2.5. Silk Fibroin

Silk is composed of fibroin (75%) and sericin (25%) [131]. Silk fibroin is a semi-crystalline structured protein and therefore has a role in load-bearing capacity, while sericin is an amorphous structured polymer [132]. For the use of tissue engineering, sericin has some unfavorable properties. First of all, sericin reduces the mechanical strength of silk fibroin fiber. The modulus of silk without sericin has about twice mechanical strength as sericin included [133]. Second, although it isn’t always necessary to be avoided, sericin sometimes induces an inflammatory response [134,135,136]. For the reasons, sericin is often removed by a degumming process under the boiling alkaline condition [137]. Silk fibroin is composed of H-chain (Mw = 391.6 kDa) and L-chain (Mw = 25.2 kDa). The two chains interact with each other by the disulfide bond, leading to the formation of the H-L complex [138]. Proteolytic enzymes, such as chymotrypsin, actinase, and carboxylase, degrade the silk fibroin. In addition, the degraded fraction does not induce an immunogenic response [139]. Due to the biocompatibility or biodegradability, silk fibroin is a useful biomaterial for the tissue engineering of bone [56,57,140], cartilage [58,59,60,141], tendon [142], skin [143,144], tympanic membrane [61], or blood vessel [145]. For example, when MSC are cultured on the silk fibroin scaffold, osteogenesis differentiation is enhanced [140]. In 2019, a silk fibroin scaffold capable of hydrogen sulfide release was prepared. This material enhances the osteogenesis of MSC, angiogenesis, or mineral matrix deposition [57]. The silk-gelatin microcarrier also achieves the osteogenic differentiation of MSC. The differentiation efficiency is comparable to that on commercial microbeads, Cultispher-S gelatin microspheres [56]. Moreover, the combination of silk fibroin and gelatin can retain the MSC, act as a physical barrier for blood clots, and provide mechanical protection of neocartilage formation [59].

### 2.6. Agarose

Agarose with a molecular weight of around 12 kDa is composed of the unit of D-galactose and 3.6-anhydro-L-galactopyranose [146]. Agarose has a capacity for water absorption, and therefore, it can permeate oxygen and nutrients to the encapsulated living cells [147]. In addition, agarose gels are formed by hydrogen bonding and electronic interaction without any harmful crosslinking agents [148]. Moreover, it has been reported that agarose doesn’t show immunogenicity [149]. Besides, the tunable properties are suitable for the application of tissue engineering because different stiffness is required depending on where it’s used. [146]. Therefore, some researchers try to use an agarose gel for the application of regenerative medicine. For example, agarose gel with the addition of polydopamine increases the water content and cell adhesion. As a result, the deposition of collagen and angiogenesis is enhanced [62]. Agarose gels containing cartilage cells with various cell seeding densities can investigate the precipitation of proteoglycan and GAG, which are the characteristics of cartilage [63]. Besides, regenerative medicine for nerve [64,65,66] or cornea [150] has been studied based on agarose.

### 2.7. Matrigel

The basement membrane comprises type Ⅳ collagen, laminin, heparan sulfate, growth factors, cytokines, or chemokines [151]. Cancer cells are attached to the basement membrane as alternative epithelial cells. Due to the basement membrane integrity, the separation between epithelial and stromal sites is achievable [152]. Cancer cells start to penetrate through the basement membrane for cancer invasion, degraded by several secreted factors, such as matrix metalloproteinase [31,153]. Therefore, the basement membrane is vital for cancer cells to enhance their biological functions. Despite the importance, human complete basement membrane can’t be constructed with current scientific technology. Therefore, as an alternative material to the basement membrane, Matrigel is often used. Matrigel is a complex protein mixture of mouse Engelbreth-Holm-Swarm tumor [32,154]. Matrigel is effective in invasion assays of cancer cells, such as Boyden chamber or transwell [155]. In addition, Matrigel is also useful for the evaluation of the morphology of cancer cells. It has been reported that there is a good relationship between the morphology of cancer cells and the profile of gene expression [156]. The combination with other biomaterials has already been studied. For example, 3D alginate and Matrigel hydrogel keep human high invasive breast cancer cells with high malignancy, spreading, migration, or invasion activities similar to those observed in vivo [67]. Furthermore, when the hydrogels are prepared by changing the mixing ratio of collagen and Matrigel, fiber diameter or pore number could be modified. This enables the evaluation of cancer cell migration into the biomimetic matrix [68]. Taken together, Matrigel is one of the most valuable biomaterials to support the culture of cancer cells with characters similar to in vivo, and Matrigel-assisted tissue engineering is also promising in cancer tissue engineering and anti-cancer drug validation among regenerative medicine.

### 2.8. Poly(lactic acid)

The elastic modulus of poly(lactic acid) (PLA) is similar to that of bone, and PLA has good thermal processability [157,158,159,160]. Therefore, PLA is used for bone tissue engineering. Significantly, the combination of hydroxyapatite (HA) and PLA is often studied because HA has an important role in ECM remodeling and homeostasis [161]. PLA-HA scaffolds, which have a porosity of more than 85%, have been prepared. The scaffolds have been used for the efficient culture of mouse embryonic osteoblasts cells because of the excellent HA distribution on the surface [69]. Zimina et al. report that the adhesion of MSC is about three times higher than that of the pure PLA sample mainly because the HA could increase the wettability of the polymeric biomaterial [70]. To scientifically support the PLA-HA scaffold, microanalysis [71] or 3D printing technology [72,73] has been recently studied.

### 2.9. Poly(lactic-co-glycolic acid)

Poly(lactic-*co*-glycolic acid) (PLGA) is a copolymer of polylactic acid and polyglycolic acid. Due to this composition, it tends to degrade more quickly than PLA. It is easy to modify the PLGA property such as degradability because the ratio of lactic acid and glycolic acid or molecular weight are the most critical factors to determine the properties [162]. In addition, PLGA formulation is prepared by simple methods, such as the solvent evaporation method or spray drying method [163]. For example, PLGA nanoparticles can be prepared by the solvent evaporation method as follows; PLGA and a hydrophobic drug are dissolved in an organic solvent, such as acetone or dichloromethane. The solution is added to aqueous poly(vinyl alcohol) solution to obtain the O/W emulsion. Then, the O/W emulsion is stirred overnight to evaporate organic solvent. The obtained particles can be directly used for various researches. Due to its biodegradability, biocompatibility, or ease of handle, PLGA is widely used in medical fields. For example, PLGA microparticles containing leuprolide are used to treat breast or prostate cancer [164]. PLGA is often selected for tissue engineering, especially for the brain or neuron [165]. Moradian et al. prepare PLGA microspheres to support the culture of neurotrophin-3 (NT-3) overexpressing cells. As a result, dopamine production and cell viability increased [78]. When MSC and nerve cells are cultured on a PLGA scaffold, both two cells proliferate and migrate. This tool is promising in the treatment of brain injury [79]. The conduit composed of PLGA promotes the Schwann cells, which stimulate axonal growth, leading to reduced cyst formation or damages [166]. In addition to the PLGA tube, the combination with salidroside promotes peripheral nerve regeneration in vitro and in vivo [167].

## 3. Conclusions and Future Perspectives

Regenerative medicine consists of the following four fields; cell transplantation, tissue engineering, drug research, and gene therapy. In each area, “utilization of cells with high activity” is essential. Therefore, the scientific methodologies to enhance cell activity contribute to regenerative medicine. In addition, although the interaction of biomaterials and targeted cells is focused in this review, the interaction of biomaterials and immune cells near the targeted cells (e.g., neutrophils or macrophages) is also an important factor because this interaction leads to the immune response. For example, macrophages are polarized to M1 (pro-inflammatory) and M2 (anti-inflammatory) phenotypes, responding to the environmental condition [168]. Therefore, when the biomaterials induce the modification of M1 macrophages, tissue regeneration would not be achieved. Moreover, the relationship between nanomaterials and immune cells has been recently investigated to understand the production of bio-corona, immune sensing, immune evasion, or degradation [169,170]. Based on these prospective, to further develop biomaterials-based regenerative medicine, the reaction of immune cells should be considered.

## Figures and Tables

**Table 1:** Recent reports on regenerative medicine combined with biomaterials.

Biomaterials	Ref.	Date	Tissue Targeted	In Vitro (Cell Type)/In Vivo (Animal Type) Testing	Results Featured
Collagen	[16]	2018	Bone	In vitro (human mesenchymal stem cells (MSC))/In vivo (mouse)	The scaffold of collagen and biphasic calcium phosphate nanoparticles with a controlled release of dexamethasone enabled the enhancement of osteogenesis from human MSC. In addition, bone regeneration was observed in nude mice.
	[17]	2019	Bone	In vitro (human MSC and human umbilical vein endothelial cells)	MSC and umbilical vein endothelial cells multicellular spheroids encapsulated in collagen/fibrin hydrogel showed efficient osteogenic differentiation.
	[18]	2020	Cartilage	In vitro (rabbit chondrocytes)/In vivo (mouse)	Porous fish collagen scaffolds promoted cartilage formation in vitro and in vivo.
	[19]	2017	Muscle	In vitro (rat skeletal myoblasts)	The 3D microgroove collagen scaffolds triggered cell assembly into anisotropic muscle bundles.
	[20]	2017	Cancer	In vitro (human breast cancer cells)	Anisotropic scaffolds supported the migration of invasive cancer cells.
	[21]	2018	Cancer	In vitro (human breast cancer cells and fibroblasts)	Tool of cancer cells and collagen gels containing fibroblasts combination system enabled the evaluation of desmoplasia, cancer proliferation, or invasion.
	[22]	2019	Cancer	In vitro (human pancreatic cancer cells, human lung cancer cells, and fibroblasts)	Cancer cells attached and migrated on the collagen matrix containing fibroblasts.
	[23]	2019	Cancer	In vitro (human breast cancer cells)	Collagen matrices with fibril bending stiffness indicated the spreading and clustering of invasive cancer cells.
Gelatin	[24]	2015	Cardiac	In vitro (human cardiovascular cell derived from iPS cells)/In vivo (mouse)	Multilayered thick cell sheets were viable by stacked with gelatin gels between each cell sheet.
	[25]	2018	Cardiac	In vivo (rat)	Basic fibroblast growth factor release from gelatin gels enabled the cell sheets to improve cardiac contractile function.
	[26]	2017	Epithelial	In vitro (mouse mammary epithelial cells and mouse preadipocyte cells)	Epithelial and preadipocyte spheroids incorporating gelatin gels promoted the expression level of laminin.
	[27]	2017	Epithelial	In vitro (mouse mammary epithelial cells)	β-casein expression was high for epithelial spheroids incorporating gelatin gels.
	[28]	2018	Pancreas	In vitro (rat insulinoma cells)	The incorporation of gelatin gels into insulinoma spheroids enabled insulin secretion.
	[29]	2018	Ovarian	In vivo (mouse)	The transplantation of gelatin sheets capable of basic fibroblast growth factor with ovarian tissues significantly increased the proliferation of stromal and endothelial cells.
	[30]	2019	Wound healing	In vivo (mouse)	Gelatin sheets impregnated platelet-rich plasma accelerated the capillary and tissue formation.
	[31]	2019	Cancer	In vitro (human lung cancer cells and fibroblasts)	A co-culture tool of cancer cells and fibroblast spheroids incorporating gelatin gels containing a p53 inhibitor can evaluate the invasion level of cancer cells.
	[6]	2020	Cancer	In vitro (human lung cancer cells and fibroblasts)	The fibroblasts spheroids incorporating gelatin gels capable of transforming growth factor-β1 increased the invasion rate of cancer cells similar to in vivo.
	[32]	2020	Cancer	In vitro (human lung, breast, and hepatic cancer cells, fibroblasts, and macrophages)	The gelatin gel-based drug release system was able to mimic the invasion ability of cancer cells, responding to the tissue region.
Alginate	[33]	2017	Germ cells	In vitro (mouse embryonic stem cells)	Alginate-collagen gels enhance primordial germ cell differentiation of embryonic stem cells.
	[34]	2020	Bone	In vitro (rat MSC)/In vivo (rat)	The osteogenesis and mineralization were observed when MSC were encapsulated into alginate gels.
	[35]	2019	Bone	In vitro (murine bone calvaria pre-osteoblast)/in vivo (mouse)	The osteoblast differentiation of pre-osteoblast was high in vitro and in vivo by encapsulating into alginate-gelatin injectable gels.
	[36]	2017	Bone	In vitro (human adipose-derived MSC)	The crosslinked oxidized alginate-gelatin hydrogel was prepared by changing the mixing ratio of alginate/gelatin. The ratio influenced osteogenic differentiation.
	[37]	2018	None	In vitro (human bone marrow-derived MSC)	Preparation of dual crosslinking homogeneous alginate microspheres combined with a microfluidics system to encapsulate MSC.
	[38]	2018	Pancreas	In vitro (human pancreatic islets)	The first trial to encapsulate human pancreatic islets in a dynamic condition, such as an organ-on-chip.
	[39]	2018	Pancreas	In vitro (mouse pancreatic β cells)/In vivo (rat)	Dual cross-linked alginate microbeads were stable under the inflammation condition in vitro and in vivo.
	[40]	2016	Cancer	In vitro (human breast cancer cells and human fibroblasts)	Alginate gels encapsulating human breast cancer cells and fibroblasts replicated phenotypic functions of cancer disease progression in vitro.
	[41]	2016	Cancer	In vitro (human umbilical cord-derived MSC and human hepatocellular carcinoma)	EMT induction or metastasis was observed when the alginate gels encapsulating hepatocellular carcinoma were co-cultured with MSC.
Chitosan	[42]	2017	Blood vessel	In vitro (human dermal fibroblast cells)	Chitosan-gelatin-based bi-layer was an appropriate scaffold to mimic the biological blood vessel, such as morphology and mechanism.
	[43]	2018	Blood vessel	In vitro (human lymphocyte cell T)	The properties of the tube showed the range value of native blood vessels (tensile strength: 2.13 MPa and burst pressure: 2593 mmHg). In addition, the tube was of high hemocompatibility and low cytotoxicity.
	[44]	2019	Blood vessel	In vitro (endothelial progenitor cells, red blood cells, or platelet-rich plasma)/In vivo (pig)	A heparin–chitosan multilayered vascular patch was biocompatible, such as a low hemolysis rate.
	[45]	2016	Cartilage	In vitro (mouse pre-chondrocytes)	The membrane of chitosan and chondroitin sulfate improved cell adhesion and enhance the expression of cartilage markers.
	[46]	2019	Cartilage	In vitro (rabbit chondrocytes)	They evaluated the mechanical and biological properties of the poly 3-hydroxybutyrate-chitosan/silk scaffold for chondrocyte viability.
	[47]	2019	Cartilage	In vitro (human cartilage)	When the graphene oxide concentration in the chitosan scaffold was high, physical and mechanical properties were improved, resulting in enhanced proliferation of chondrocytes.
	[48]	2017	Cartilage	In vitro (mouse pre-chondrocytes)	Preparation of chitosan/poly(vinyl alcohol)/graphene oxide nanofiber for cartilage tissue engineering.
	[49]	2016	Bone	In vitro (human bone osteosarcoma cells)	Chitosan-montmorillonite-hydroxyapatite composite scaffolds were non-cytotoxic, and the properties, such as bioactivity or protein absorption, were improved compared with chitosan or chitosan-montmorillonite scaffolds.
	[50]	2017	Bone	In vitro (human bone marrow-derived MSC)	Chitosan nanohybrid combined with strontium hydroxyapatite enhanced osteoconductivity.
	[51]	2017	Intervertebral disc	In vitro (rabbit nucleus pulposus cells from lumbar disc)	Chitosan-based injectable gels indicated constant storage modulus similar to the intervertebral disc ECM.
	[52]	2019	Intervertebral disc	In vitro (bovine nucleus pulposus cells from coccygeal intervertebral disc)	Thermosensitive chitosan hydrogels with high strength and rheological properties were prepared.
	[53]	2019	Intervertebral disc	In vitro (rabbit nucleus pulposus cells and annulus fibrosus cells)/In vivo (rabbit)	Preparation of chitosan hydrogel/poly (butylene succinate-co-terephthalate) copolyester electrospun fibers for intervertebral disc therapy.
	[54]	2017	Skin	In vitro (mouse fibroblast cells)	Electrospun multilayer chitosan scaffolds with low cytotoxicity were prepared. The scaffolds have high porosity, and the mechanical properties of the scaffolds matched those of the human skin.
	[55]	2019	Skin	In vitro (mouse fibroblast cells)	The chitosan-vitamin C scaffolds with glycerol and polyethylene glycol enhanced the activity of skin cells.
Silk fibroin	[56]	2020	Bone	In vitro (rat bone marrow-derived MSC)	They evaluated the appropriate mixing ratio of silk fibroin/gelatin as a microcarrier for efficient osteogenic differentiation.
	[57]	2019	Bone	In vitro (human bone marrow-derived MSC)	Hydrogen sulfide-releasing silk fibroin scaffolds induced osteogenesis.
	[58]	2016	Cartilage	In vitro (porcine chondrocytes)/In vivo (rat)	When the chondrocytes were cultured on the silk fibroin scaffolds of *Antheraea assamensis*, sulfated glycosaminoglycans and type Ⅱ collagen production increased.
	[59]	2017	Cartilage	In vitro (rat bone marrow-derived MSC)/In vivo (rabbit)	They optimized the mixing ratio of silk fibroin to gelatin as scaffolds prepared using 3D printing for cartilage repair.
	[60]	2016	Cartilage	In vitro (pig auricular chondrocytes)	The combination of agarose and silk fibroin enhanced the polymeric network, leading to the up-regulation of cartilage-specific genes.
	[61]	2016	Tympanic membrane	In vitro (pig cartilage)	The first report on the effect of silk fibroin membranes on the acoustic energy transfer and tensile strength to cartilage.
Agarose	[62]	2021	Skin	In vitro (human normal embryonic lung fibroblast cells)/In vivo (mouse)	Agarose-polydopamine hydrogels were biocompatible scaffolds capable of promoting collagen deposition and angiogenesis, finally skin defect healing.
	[63]	2017	Cartilage	In vitro (human elastic cartilage-derived chondrocytes)	Nanostructured fibrin–agarose hydrogel enabled chondrocytes encapsulation and support of culture.
	[64]	2019	Nerve	In vitro (rat neuronal cells)	Electrical stimulation facilitated dexamethasone release from hydrogels.
	[65]	2017	Nerve	In vitro (rat adipose-derived MSC)/In vivo (rat)	Collagen conduits filled with fibrin–agarose hydrogels containing stem cells were prepared for nerve regeneration.
	[66]	2017	Nerve	In vitro (human adipose-derived MSC)	A nanostructured fibrin-agarose bioartificial nerve substitute enabled stem cells to proliferate.
Matrigel	[67]	2018	Cancer	In vitro (human breast cancer cells)	The cancer cell-laden gels composed of the appropriate mixing ratio of Matrigel and alginate replicate the behavior of cancer cells.
	[68]	2017	Cancer	In vitro (human non-small cell lung carcinoma)	Matrigel and collagen-based microfluidics systems can control the migration of cancer cells by changing the Matrigel concentration.
Poly(lactic acid) (PLA)	[69]	2019	Bone	In vitro (mouse embryonic osteoblast cells)	The attachment and proliferation of cells on poly(lactic acid)-hydroxyapatite (HA) hybrid scaffolds increased. The result is mainly because of the interaction between cells and scaffolds via HA.
	[70]	2020	Bone	In vitro (cat bone marrow-derived MSC)/In vivo (mouse)	PLA-HA improved the adhesion of cells, and widespread ingrowth of tissues into the implant pores was observed.
	[71]	2020	Bone	None	Microanalysis of PLA-HA scaffolds was performed.
	[72]	2021	Bone	In vitro (human fetal osteoblast cells)	PLA-based scaffolds provided porous networks and gave cells good biological functions, such as osteogenesis.
	[73]	2021	Bone	In vitro (rabbit MSC)/In vivo (rabbit)	PLA scaffolds incorporating a high concentration of HA showed efficient bone regeneration.
Poly(lactic-*co*-glycolic acid) (PLGA)	[74]	2018	Bone	In vitro (human osteosarcoma cells)/In vivo (rabbit)	The amount of bone formation for TiO_2_ nanotube/PLGA scaffolds was much higher than for PLGA scaffolds.
	[75]	2021	Bone	In vitro (human adipose or bone marrow-derived MSC)/In vivo (rat)	PLGA-hydroxyapatite (HA) nanoparticles promoted osteodifferentiation compared to the PLGA scaffold.
	[76]	2019	Cartilage	In vitro (rabbit synovium-resident MSC)/In vivo (rabbit)	Bone morphogenetic proteins-7 loaded fibrous PLGA scaffolds combined with MSC showed a cartilage formation.
	[77]	2020	Cartilage	In vitro (rabbit bone marrow-derived MSC and rabbit chondrocytes)	When cells were cultured on insulin-like growth factor-1 laden PLGA/polydopamine/poly-ε-caprolactone scaffolds, glycosaminoglycan content, chondrogenic protein, and gene expression increased.
	[78]	2017	Nerve	In vitro (rat bone marrow-derived MSC)	PLGA microcarriers were promising scaffolds to support the culture of neurotrophin-3-overexpressing stem cells.
	[79]	2018	Nerve	In vitro (rat bone marrow-derived MSC and rat cortical neurons)	Stem cells and neurons could grow and migrate in the PLGA scaffolds.
